# Investigating the Impact of Brief Outings on the Welfare of Dogs Living in US Shelters

**DOI:** 10.3390/ani11020548

**Published:** 2021-02-19

**Authors:** Lisa M. Gunter, Rachel J. Gilchrist, Emily M. Blade, Rebecca T. Barber, Erica N. Feuerbacher, JoAnna M. Platzer, Clive D. L. Wynne

**Affiliations:** 1 Department of Psychology, Arizona State University, Tempe, AZ 85281, USA; rgilchri@asu.edu (R.J.G.); eblade@asu.edu (E.M.B.); cwynne1@asu.edu (C.D.L.W.); 2 Division of Education Leadership and Innovation, Arizona State University, Tempe, AZ 85281, USA; rebecca.barber@asu.edu; 3 Department of Animal and Poultry Sciences, Virginia Polytechnic Institute and State University (Virginia Tech), Blacksburg, VA 24061, USA; enf007@vt.edu (E.N.F.); jplatzer@vt.edu (J.M.P.)

**Keywords:** dogs, animal shelter, cortisol, stress, welfare, human-animal interaction, activity

## Abstract

**Simple Summary:**

Animal shelters can be stressful places for dogs to live. Social isolation is likely one component of the environment that contributes to poor welfare but spending time out of the kennel with a person has been shown to temporarily ameliorate that stress. In this study, 164 shelter-living dogs at four animal shelters across the United States were taken on two-and-half-hour outings with a person and physiological measures of stress and physical activity captured by accelerometer devices were compared before, during, and after this short-term outing. We found that dogs’ stress was higher when they were away on these field trips and their activity changed, including less time spent in low activity and more time in higher activity. While measures of physiology and activity were found to return to pre-field trip levels the following day, these results suggest that outings of this duration do not provide the same reduction in stress as previously shown with temporary fostering. Nevertheless, short-term outings may provide shelter dogs with greater adoption visibility and assist in foster recruitment and, thus, should be further explored.

**Abstract:**

Social isolation likely contributes to reduced welfare for shelter-living dogs. Several studies have established that time out of the kennel with a person can improve dogs’ behavior and reduce physiological measures of stress. This study assessed the effects of two-and-a-half-hour outings on the urinary cortisol levels and activity of dogs as they awaited adoption at four animal shelters. Dogs’ urine was collected before and after outings for cortisol:creatinine analysis, and accelerometer devices were used to measure dogs’ physical activity. In total, 164 dogs participated in this study, with 793 cortisol values and 3750 activity measures used in the statistical analyses. We found that dogs’ cortisol:creatinine ratios were significantly higher during the afternoon of the intervention but returned to pre-field trip levels the following day. Dogs’ minutes of low activity were significantly reduced, and high activity significantly increased during the outing. Although dogs’ cortisol and activity returned to baseline after the intervention, our findings suggest that short-term outings do not confer the same stress reduction benefits as previously shown with temporary fostering. Nevertheless, it is possible that these types of outing programs are beneficial to adoptions by increasing the visibility of dogs and should be further investigated to elucidate these effects.

## 1. Introduction

Between 4.0 and 5.5 million dogs enter animal shelters each year in the US [1,2]. Considerable efforts have been made over the past two decades to improve outcomes for dogs facing this experience (for a review, see [3]), leading to more dogs being adopted and returned to their owners and reduced levels of euthanasia [2,4].

More recently, animal welfare organizations have begun focusing on the standard of care that dogs receive while in the shelter [5]. In part, this is in recognition of the potential stressors within the environment, including excessive noise [6,7,8,9], spatial restriction [10,11,12], social isolation [13], loss of attachment figures [14], loss of control [15] and lack of a daily routine [16]. One way to mitigate the impact of these stressors is through the use of enrichment interventions intended to improve welfare [17,18,19].

The most commonly studied enrichment intervention in sheltering is interactions with people [15,16,20,21,22,23,24,25,26,27,28,29,30,31,32]. The majority of these interventions occur at the shelter but out of the kennel and are 15–45 min in duration. Often, their impacts are measured by changes in physiology and behavior.

Cortisol is one of the most widely utilized physiological markers of stress in dogs [33]. Previous studies have found elevated cortisol levels for dogs living in shelters as compared to those in homes [29,34] as well as for dogs from homes entering kennels for the first time [35]. Resting activity or sleep can also be a useful component in welfare assessment. Dogs in shelters have been observed to sleep less during the day than dogs in homes [36,37] and had more activity during their most and least active hours than owned dogs [38], suggesting a lack of restful activity for shelter dogs.

A handful of human-interaction interventions, such as those by Hennessy et al. [22], Gunter et al. [32], and Fehringer [28], describe dogs leaving the animal shelter which may provide greater relief to dogs than in-shelter interactions. Gunter et al. [32] reported the impacts of one- and two-night stays in volunteers’ homes and measured dogs’ cortisol levels before, during, and after those stays. Dogs were found to have lower cortisol levels in homes; and while dogs’ cortisol increased upon return to the shelter, it was no higher than baseline levels. Additionally, dogs’ bouts of uninterrupted rest were longest when in the foster home, but still remained longer upon return to the shelter than prior to fostering. Fehringer [28] also found that placement in a home resulted in lower cortisol compared to in-shelter levels, and dogs’ cortisol steadily declined over the first three days in foster care.

Little is known about how short-term outings of a few hours in duration without an overnight stay could impact the welfare of dogs awaiting adoption. Considering that previously tested in-shelter interventions of less than one hour have been shown to reduce cortisol and improve behavior [15,16,21,26,27,29,30,31], it is possible that out-of-shelter outings of a slightly longer duration could confer even greater benefits. However, it is worth noting that many of these aforementioned interventions took place in living room-like settings at the shelter and involved calm interactions with the dog lying down and being petted by a person.

Conversely, short-term outings into the community such as field trips allow for increased physical activity for the dog. Yet a prior study failed to find clear evidence of behavioral improvement following physical activity when provided to dogs at the shelter. Protopopova, Hauser, Goldman, and Wynne [39] compared interventions of exercise and reading offered for 15 min daily for two weeks. Both interventions were followed by increases in dogs’ back-and-forth motion in the kennel, a locomotor behavior associated with reduced welfare and longer lengths of stay [40]. While a greater reduction in door jumping was observed after exercise as compared to the reading intervention, dogs were more often at the front of their kennels and barking less often after volunteers read to them for the same duration. Thus, it seems likely that the activity a person engages in with a shelter dog could differentially affect its welfare.

In the present study, we explored whether short-term outings with a person away from the animal shelter would influence dogs’ urinary cortisol:creatinine (C/C) ratios in the afternoon of the intervention as compared to ratios collected in the shelter before and after these outings. Additionally, dogs’ physical activity was monitored throughout the study to detect potential differences in activity intensity.

## 2. Materials and Methods

### 2.1. Shelters

Data were collected at four animal shelters in the United States: Spokane County Regional Animal Protection Service (SCRAPS, June 2019); Fulton County Animal Services in Atlanta, GA (FCAS, July 2019); the Regional Center for Animal Care and Protection in Roanoke, VA (RCACP, August and October 2019); and Detroit Animal Care and Control (DACC; September 2019). The shelters varied in their geographical location and annual dog intake but all were open-admission facilities (Table 1).

These shelters had existing short-term outing programs prior to data collection, although their weekly usage varied, ranging from one or less at RCACP to five or less per week at SCRAPS, FCAS, and DACC. Additionally, DACC held monthly events in which typically sixty-five or more dogs would leave on short-term outings with shelter volunteers. Data collection at DACC began 19 days after their last event.

In addition to providing short-term outings, all shelters in this study had walking programs for their dogs in which dogs would spend time out of their kennels interacting with volunteers in and around the facility. Both FCAS and DACC had temporary fostering programs in which dogs would leave the shelter for stays in a caregiver’s home. Dogs at all four shelters had access to elevated, cot-style beds in their kennels and also received sporadic food enrichment. At SCRAPS and FCAS, dogs interacted with each other outdoors in supervised groups.

Staff at each of the facilities determined which dogs would participate in this study. To minimize the likelihood of injury or harm to individuals carrying out these field trips, shelters selected dogs without histories of aggressive behavior. Dogs enrolled in the study at FCAS and RCACP had not previously experienced an outing, while it is possible that dogs at SCRAPS and DACC that entered the shelter prior to data collection may have done so. Dogs that were fearful during urine collection were not included due to the researchers’ inability to obtain samples for cortisol analysis.

### 2.2. Short-Term Outings

Dogs experienced approximately two-and-a-half-hour-long outings with a person between 11:00 a.m. and 3:00 p.m., off the property of the animal shelter. (Less than two percent of outings occurred outside of these times.) Volunteers, staff, and members of the public were eligible to take dogs on outings but were required to meet organizational requirements prior to participation, including being over 18 years of age and providing a driver’s license for identification. Shelter staff provided participating individuals with handling instructions and supplies, and the authors discussed the purpose of this study. When dogs returned to the shelter, the research team asked these individuals to complete a questionnaire about their outing, and dogs were placed back into their kennels.

### 2.3. Collection Timeline

Study enrollment lasted three days for each dog with five collections per dog. Beerda et al. [12] found that both urinary and salivary cortisol levels of kenneled dogs were higher in the mornings than in the evenings. However, in studies in which working dogs were active in both the morning and evening as compared to dogs that only had daytime activities, this across-the-day reduction in cortisol was not present [41]. Given this uncertainty regarding the presence of a circadian influence with the cortisol levels of shelter dogs, collections occurred in both the morning and afternoon to capture potential changes over time as well as those caused by the outing.

Morning collection on Days 2 and 3 was conducted between 7:00 a.m. and 9:30 a.m. These times are consistent with those used by Gunter et al. [32]. Afternoon collections on Days 1–3 occurred between 3:30 p.m. and 6:00 p.m. Table 2 provides the experimental timeline for collection. Less than one percent of samples fell outside these collection windows due to dogs not urinating when walked or providing an inadequate volume of urine (minimum 1.5 mL). In these cases, dogs were provided a mixture of wet food and water and walked until the dog urinated.

### 2.4. Urine Collection

The research team collected dogs’ urine before and after their outings for C/C analysis. Dogs were removed from their kennels on leash and walked to locations outside of the shelter for urine collection and returned to their kennels after samples were obtained. Olympic Clean-Catch™ plastic trays taped to 36 inch (91 cm) “Pickup and Reach” tools (Harbor Freight, Calabasas, CA, USA) were used for urine collection. Samples were poured from the collection trays into 10 mL plastic tubes with snap caps for storage. Trays were rinsed with water and air-dried or wiped with sterile KimWipes™ (Kimberly-Clark, Irving, TX, USA) between collections. Samples were immediately placed in a cooler with ice after collection, and in a freezer within two hours at a temperature of −18 °C.

Frozen urine samples were shipped overnight on dry ice to ZNLabs Veterinary Diagnostics (Louisville, KY, USA) for C/C analysis. Analysis was conducted using an automated wet biochemistry analyzer (AU680, Beckman Coulter, Brea, CA, USA) for measurement of creatinine. Bio-Rad Liquid Human Urine Precision Chemistry Controls 1 and 2 (Bio-Rad Laboratories, Inc., Hercules, CA, USA, Control Level 1 #397, Control Level 2 #398) were run on each day of urine sample testing and stored according to manufacturer instructions. Cortisol was measured using a commercially available product designed for an enzyme-amplified chemiluminescence assay system (Immulite 2000 XPi, Siemens Healthcare Diagnostics, Inc., Newark, DE, USA). Cortisol:creatinine ratios (measured in μmol/L: μmol/L) × 10^−6^ were then calculated.

### 2.5. Activity Monitoring

Whistle FIT activity monitors (Whistle Lab Inc., San Francisco, CA, USA) were attached to collars and placed on the dogs, allowing for collection of their movement via the triaxial accelerometer. Placement of collar-mounted monitors occurred during the morning of the dogs’ first day in the study and worn for the study’s duration, unless battery loss or malfunction resulted in removal and replacement with a new device. Data from the Whistle FITs were transmitted to Whistle servers via each shelter’s wireless network and Bluetooth.

Yashari, Duncan, and Duerr [42] assessed the validity of Whistle devices as a measure of canine activity by comparing data generated from these devices with that of Actical, a previously validated activity monitor. They found that measurements of dogs’ activity intensity and total activity were highly correlated between the two devices, 0.81 and 0.93, respectively.

For the purposes of this study, dogs’ activity was calculated using the raw data recorded by the Whistle devices. These triaxial accelerometers collected the x, y, and z components of a vector representing dog movement, *M* = (*M_x_, M_y_, M_z_*), at a rate of 50 times per minute. The magnitude of this vector, *M = √ (M_x_^2^, M_y_^2^, M_z_^2^)*, was used to indicate dogs’ composite movement at each time period. Magnitude calculations were then summed over one-minute epochs as an estimate of the dogs’ activity during that minute. Magnitude-per-minute values ranged from 0.89 to 8102.

To characterize this activity, all magnitude-per-minute (m/m) values were categorized into one of five, evenly apportioned activity levels. Quintile thresholds were derived from the complete set of magnitude values obtained throughout the study. Each quintile thus contains approximately 37,576 records. The magnitude-per-minute thresholds and associated activity categories, Q1 (lowest) through Q5 (highest), are shown in Table 3.

M/m values were calculated for the four hours prior to the five urine sample collections, a time window based on the previously demonstrated reflection period of canine urinary cortisol [43]. Dogs’ total minutes in each activity level as well as the proportion of time spent in each of those levels were calculated. Data from dogs with at least 200 m/m values for the four hours prior to the urine collection were used in our analysis.

### 2.6. Statistical Analysis

To investigate whether dogs’ cortisol differed across time, by shelter or in a shelter-by-timepoint interaction, we analyzed C/C ratios obtained for the dogs at our four study sites with a linear mixed model.

Dog and intercept were entered as random effects with timepoint, shelter, and a timepoint-by-shelter interaction along with the covariates of age, weight, length of stay (LOS), and the activity categories included as fixed effects. These covariates were included in the model as they have been previously found to influence cortisol [32]. A variance covariance matrix was employed, and a diagonal covariance matrix for the repeated measure of timepoint. The method of Restricted Maximum Likelihood (REML) was used for estimating parameter values.

To explore whether the number of minutes dogs spent in the five activity categories changed throughout this study, by shelter or in a timepoint-by-shelter interaction, we performed a doubly multivariate analysis with a general linear model. Dogs’ weight, age, and mean LOS (across the three days of the study) were entered as covariates. For the within-subjects variable of time, the contrast was polynomial and for shelter, simple.

With both models, dogs’ age and LOS were log transformed to ensure the normal distribution of variables. When post-hoc comparisons were conducted in our analyses, a Sidak correction was utilized to reduce the likelihood of false positives when multiple comparisons were made. A statistical significance level of *p* < 0.05 was used throughout.

### 2.7. Ethical Statement

Procedures carried out at SCRAPS, FCAS, DACC, and RCACP were approved by the Arizona State University Institutional Animal Care and Use Committee (IACUC: 17-1552R).

## 3. Results

### 3.1. Descriptive Statistics

When characterizing the dogs at SCRAPS, FCAS, DACC, and RCACP, they were more often male (56.1%), with most dogs arriving to the shelter as a stray (76.2%). Nearly one-fifth of dogs (19.5%) were surrendered by their owners or returned after a failed adoption. On average, dogs were slightly over three years of age (*M* = 39.00 months, *SD* = 30.96) and weighed 23.96 kg (*SD* = 7.55). The number of days dogs were living in the shelter at the time of the study ranged from 1.50 to 252.50 days (*M* = 38.95, *SD* = 42.45).

In total, 164 short-term outings were conducted as part of this study, with 40 dogs participating at FCAS, 41 each at both SCRAPS and DACC, and 42 at RCACP. In an effort to better understand what transpired during these outings, we collected information about the individuals that provided these outings as well as the activities they engaged in with the dogs and where those activities occurred.

Nearly two-fifths of individuals that took dogs on short-term outings were public participants (37.80%), with the remaining individuals being shelter staff and volunteers or members of the research team, and most often those that were providing outings were female (86.59%). Over half of all outings (51.83%) included just one person and the shelter dog. Over one-third of short-term outings (35.37%) did not involve any additional people interacting with the dog, such as petting, playing, or offering the dog a treat, while more than half (53.05%) involved additional interactions with 1–5 people. Shelters varied by the individuals taking dogs on these outings and their interactions (Table 4).

Over three-quarters of dogs (75.60%) spent time outdoors on their field trip, such as visiting a park, where they walked, hiked, or jogged with the person, and almost 30% of dogs (29.90%) visited a pet-friendly store or restaurant in the community. Fewer than half of the dogs (43.90%) visited the person’s home while on the field trip, with only 22.00% of dogs exclusively spending time in a home during the outing.

### 3.2. Cortisol Analysis

Dogs at SCRAPS, FCAS, DACC, and RCACP yielded 793 cortisol values that were statistically analyzed across the five urine collections in this study to detect an effect of time of collection, shelter, or shelter-by-timepoint interactions with dogs’ weight, (log) age, (log) LOS and minutes spent in each activity category added into the model as covariates. 

With this model, the variables of shelter, timepoint, shelter-by-timepoint interaction, weight, and log (LOS) were significant (at *p* < 0.05), with log(age) marginally significant at *p* = 0.061. None of the activity categories were statistically significant but were retained in the model to account for the effect of activity on C/C ratios.

**Figure 1:**
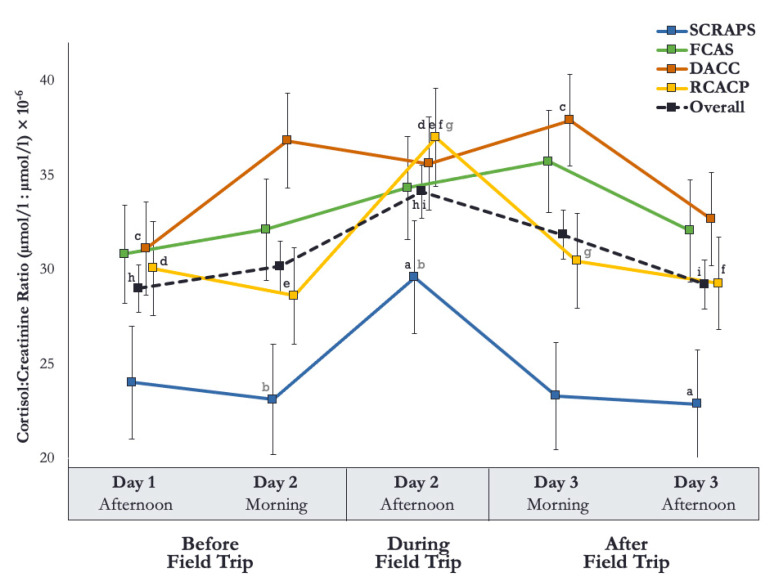
Estimated marginal means of dogs’ cortisol:creatinine ratio values and standard errors for the five study timepoints by shelter. “Overall” represents the estimated marginal means and standard errors at each timepoint, regardless of shelter. All comparisons (shared letters: a–i) are significant at *p* < 0.05, except for comparisons b (*p* = 0.051) and g (*p* = 0.068).

The main effect of timepoint tested was significant, *F* (4, 560.42) = 6.29, *p* < 0.001, demonstrating that the dogs’ cortisol changed across the study. In post-hoc comparisons, dogs were found to have significantly higher cortisol values on the afternoon of the field trip as compared to the afternoon of the day before (*p* < 0.001) and the afternoon of the day after (*p* = 0.001). Figure 1 presents the estimated marginal means and standard errors of the cortisol values for the five timepoints across the three days of the study.

A main effect of shelter was also detected, *F* (3, 149.15) = 3.19, *p* = 0.026, revealing that the estimated marginal means for cortisol varied amongst the shelters. In post-hoc comparisons, dogs at SCRAPS had the lowest C/C ratios, which were significantly different from dogs at DACC (*p* = 0.017). Table 5 includes the average estimated marginal means of C/C ratios and standard errors for the dogs at SCRAPS, FCAS, DACC, and RCACP. When this analysis was repeated, excluding cortisol values from the afternoon of the field trip to examine only pre- and post-intervention timepoints in the shelter, this effect was slightly more pronounced, *F* (3, 149.79) = 4.00, *p* = 0.009. Post-hoc comparisons and subsequent differences between dogs at SCRAPS and those at DACC (*p* = 0.007) were marginally greater.

The interaction of shelter-by-timepoint was significant, *F* (12, 537.12) = 2.01, *p* = 0.022, indicating that dogs’ cortisol values differed at each of the shelters at the various study timepoints. When examining these shelter-specific timepoint differences, dogs at SCRAPS had significantly higher cortisol values on the afternoon of the field trip as compared to the following afternoon (*p* = 0.043) and marginally so the morning before the field trip (*p* = 0.051). Dogs at DACC had significantly higher cortisol on the morning of the day following the field trip as compared to the afternoon of the day before the field trip (*p* = 0.020). At RCACP, dogs had significantly higher cortisol during the afternoon of the field trip as compared to the afternoon of the day before the field trip (*p* = 0.020), the morning prior to the field trip (*p* = 0.007), the afternoon of the day after the field trip (*p* = 0.005), and marginally so the morning after the field trip. Figure 1 presents the estimated marginal means and standard errors of the cortisol values at each shelter across the study timepoints.

When investigating differences between shelters at the same collection timepoint, dogs at DACC had significantly higher morning cortisol values on the days pre- and post-field trip than dogs at SCRAPS (*p* = 0.004 and *p* = 0.001, respectively). The morning values post-field trip on Day 3 were also significantly higher at FCAS (*p* = 0.017) and marginally so at DACC (*p* = 0.064) than values obtained at SCRAPS. Table 5 includes the estimated marginal means of C/C ratios and standard errors at each collection timepoint at SCRAPS, FCAS, DACC, and RCACP.

### 3.3. Activity Analysis

At SCRAPS, FCAS, DACC, and RCACP, 121 dogs provided 710 readings of minutes spent in each of the five activity categories. Minutes were analyzed across the five study timepoints to detect an effect of time, shelter, or a shelter-by-timepoint interaction with dogs’ weight, (log) age, (log) meanLOS entered as covariates in the model.

With this model, Box’s Test of Equality of Covariance Matrices was shown to be violated (*p* < 0.001), indicating that the covariance matrices of the dependent variables were not equal. As such, test statistics are reported here are using Pillai’s Trace as it is considered to be the most robust test to violations of model assumptions [44]. Mauchly’s Test of Sphericity was also violated for four of the five activity categories (*p* < 0.001), with the exception of activity category Q3 (*p* = 0.369), thus sphericity was not assumed, and Greenhouse–Geisser tests were used to determine statistical significance.

**Figure 2:**
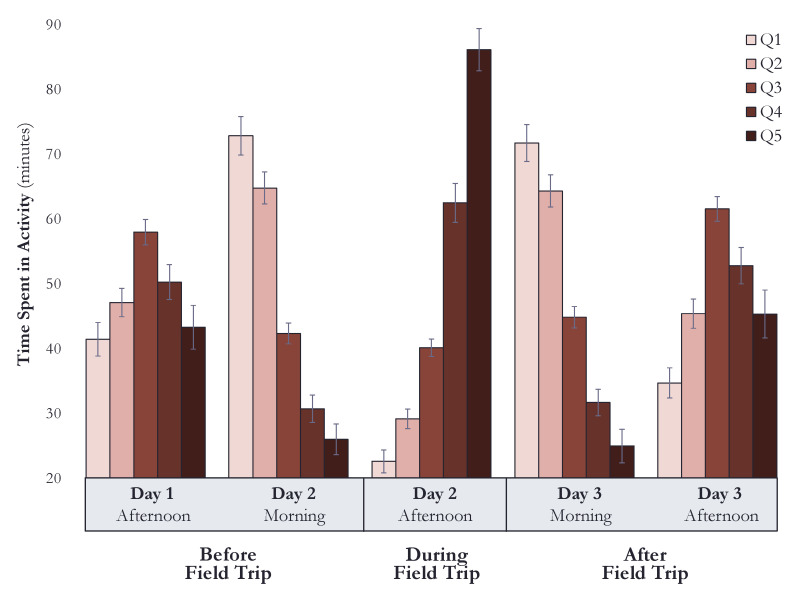
Estimated marginal means and standard errors of minutes spent in the five activity categories during the four hours prior to each of the five urine collection timepoints.

We found that timepoint significantly influenced dogs’ minutes in the five activity categories, *F* (20, 95) = 41.78, *p* < 0.001, demonstrating that their activity varied across the three days of this study. In post-hoc comparisons, dogs spent less time in the lower activity categories of Q1 and Q2 during the afternoon of the field trip than any other time in the study (*p* < 0.001). Conversely, dogs spent significantly more time in the higher activity categories, Q4 and Q5, during the afternoon of the field trip than all other timepoints (*p* < 0.001). Figure 2 presents the estimated marginal means and standard errors of minutes spent in the five activity categories for the five timepoints across the three days of the study.

A significant effect of shelter was also found, *F* (15, 336) = 2.20, *p* = 0.006, indicating that the minutes dogs spent in the various activity categories differed amongst the shelters; however, in post-hoc comparisons, only one difference was found between shelters. Dogs spent more time in Q3 activity at DACC than dogs at RCACP (*p* = 0.007).

A significant shelter-by-timepoint interaction was detected, indicating that the time dogs spent in each of the activity categories differed between shelters at the various study timepoints. (SCRAPS: *F* (20, 95) = 8.91, *p* < 0.001; FCAS: *F* (20, 95) = 5.97, *p* < 0.001; DACC: *F* (20, 95) = 12.53, *p* < 0.001; RCACP: *F* (20, 95) = 15.88, *p* < 0.001.) When examining shelter-specific activity levels, two patterns were apparent. Firstly, activity during the afternoon of the field trip was generally different from other timepoints; and secondly, the activity recorded in the mornings and afternoons differed from each other, mirroring the timepoint post-hoc comparisons previously reported (see Figure 2). One additional difference was seen at SCRAPS, where dogs spent less time in the lowest activity category (Q1) during the afternoon of the day after the field trip than the afternoon of the day before the field trip (*p* = 0.018).

When exploring whether dogs’ activity varied across study timepoints at the shelters, the timepoint-by-shelter interaction was significant, with the exception of the morning of the day after the field trip, indicating that minutes of time spent in the various activity categories at each of the study timepoints differed by shelter. (Timepoints 1: *F* (15, 336) = 2.39, *p* = 0.003; 2: *F* (15, 336) = 1.54, *p* = 0.088; 3: *F* (15, 336) = 2.18, *p* = 0.007; 4: *F* (15, 336) = 1.41, *p* = 0.138; 5: *F* (15, 336) = 2.10, *p* = 0.003.) We found that dogs at SCRAPS had more low activity (Q1) than dogs at DACC during the afternoon of Day 1 (*p* = 0.034), fewer Q1 minutes in the morning of Day 2 as compared to the dogs at DACC (*p* = 0.036) and RCACP (*p* = 0.013). Dogs at RCACP also had significantly more minutes of Q1 low activity during the afternoon of Day 3 than dogs at DACC (*p* = 0.004).

When exploring the second-lowest activity category (Q2), the only differences detected were during the afternoon of the field trip: dogs at FCAS had more Q2 activity than dogs at SCRAPS (*p* = 0.039) and RCACP (*p* = 0.048). With regards to moderate Q3 activity, DACC dogs spent more time in this category during the first afternoon of this study than dogs at FCAS (*p* = 0.001) and RCACP (*p* = 0.007) and marginally more time than RCACP dogs during the morning of Day 3 (*p* = 0.054). Only two differences were found in dogs’ higher activity (Q4), and those were between the same shelters in the mornings before and after the field trip: dogs at SCRAPS had more higher activity than dogs at RCACP (*p* = 0.008 and *p* = 0.002, respectively). Lastly, dogs at RCACP spent more time in the highest activity (Q5) during the field trip than dogs at FCAS (*p* = 0.001).

## 4. Discussion

In our investigation of short-term outings, we found that this intervention increased the stress of shelter dogs and decreased their resting activity. Even after accounting for activity in our cortisol analysis, we found that dogs’ C/C values were significantly higher during the afternoon of intervention as compared to the prior and following afternoons. However, cortisol did return to pre-outing levels by the next day. Additionally, we found that dogs of greater weight had lower cortisol values, and older dogs had higher cortisol values (as previously shown by Zeugswetter et al. [45] and Rothuizen et al. [46], respectively). These new findings support the previously reported relationships of dogs’ weight and age to cortisol [32]. Dogs with shorter lengths of stay also had higher cortisol values than those with longer stays in the shelter.

In our analysis of time dogs spent in the five activity levels across this study, we found similar apportioning of activity in the mornings and afternoons prior to and after field trips. During the mornings, dogs were spending the most time in low activity (see Table 3). Not surprisingly, these are early morning hours prior to staff arrival when dogs are often sleeping, supporting prior findings about the activity of dogs in shelters [36,38]. Conversely in the afternoons pre- and post-outing, dogs were more active than the morning, spending more minutes in the higher and mid-activity levels of Q5, Q4, and Q3.

### 4.1. Duration, Activities, and Location of Human Interaction with Shelter Dogs

To our knowledge, the present study is the first investigation of an intervention in which dogs leave the shelter for a few hours and then are returned to their kennels. More commonly in the scientific literature, human interaction is provided to dogs while remaining at the shelter. In these studies [15,16,21,26,27,29,30,31], interactions with the person ranged from 15 to 45 min in duration and dogs’ cortisol was found to temporarily decrease, a finding that was not replicated in the present study despite the increased duration (2.5 h) of human interaction.

It may not be the duration of the interaction, though, that is as important as the location and activities undertaken between the person and the dog. In the aforementioned studies where the dogs’ cortisol levels were lower following the intervention, particularly those by Hennessy et al. [15], Hennessy et al. [16], Shiverdecker et al. [27], Dudley et al. [29], Willen et al. [30], and McGowan et al. [31], dogs were removed from their kennels and the interaction occurred at the shelter but in a quiet, secluded room. In the present study, less than one-quarter of the dogs returned to the person’s home for their outing, while the majority of dogs spent time walking, hiking, or jogging in public. When Gunter et al. [32] and Fehringer [28] reported reductions in cortisol in their fostering interventions, dogs were taken to homes for one and two or three nights, respectively.

When examining dogs’ activity across the three days of this study, dogs were more active in the greater intensity categories during the short-term outings. Specifically, they spent nearly twice as long in Q5 activity as any other afternoon in the study. Previous research by Radosevich et al. [47] demonstrated that as the intensity and duration of exercise increased so did dogs’ cortisol. This arousing effect of activity on cortisol has also been found with sled dogs [48,49] and dogs off leash at a dog park [50].

Contrariwise, cortisol reductions observed by Hennessy et al. [15], Hennessy et al. [16]; Shiverdecker et al. [27], Dudley et al. [29], Willen et al. [30], and McGowan et al. [31] were induced after dogs received calm petting, with the person even massaging and talking soothingly to the dog during the intervention (with the exception of the stranger and play conditions in Shiverdecker et al. [27]). Similarly, Gunter et al. [32] reported a relaxing experience in the home, with dogs having their longest bouts of uninterrupted rest during temporary fostering.

### 4.2. Psychological Stress and Increases in Cortisol

Based on the statistical analysis, however, activity alone does not explain this rise in cortisol during the afternoon of the outing, allowing for the possibility that the difference in cortisol was related to the psychological stress of the field trip. Instead of a quiet room where dogs could escape the stressors of the shelter or spend time resting in a home, dogs were active on these field trips and exposed to a variety of novel sights and sounds. If visual, auditory, and olfactory stimuli in the shelter have been found to negatively impact dogs’ welfare [17], field trips that include activities such as outdoor dining, hiking, or visiting a store could be stressful, too. Future studies, where volunteers exclusively take dogs to homes or other quiet locations during the outings and calmly interact with them instead of partaking in more energetic activities may find the type of reduction in cortisol that was reported in previous studies.

Increases in cortisol, as reported here with short-term outings, do not necessarily indicate poorer welfare for dogs. Owned dogs engage in a variety of preferred activities that positively impact their welfare and are accompanied by higher cortisol levels, such as attending the dog park [50], competing in agility [51] or hunting [52]. Certainly, these activities are arousing; but we propose that it is the environments in which these dogs are living that should be considered. After such activities, owned dogs return to their homes while dogs awaiting adoption return to the stressful environment of the shelter. Zeugswetter and colleagues [45] identified that the median morning C/C ratio for healthy owned dogs is just 16 (μmol/L: μmol/L × 10^−6^), while in the present study, the median cortisol value in the morning was 27.1 (μmol/L: μmol/L × 10^−6^).

### 4.3. Proximate Welfare Interventions

Our research and that of others [29,34] indicate that shelter dogs have highly elevated cortisol levels, and these levels likely persist for a prolonged period of time [18]. In addition to physiological measures of stress, shelter dogs spend significantly less time resting than dogs living in homes [38]. By all accounts, it would seem that instead of further arousing dogs that are already coping with an unpredictable environment, enrichment interventions should be aimed at reducing dogs’ cortisol levels and other physiological and behavioral measures indicative of compromised welfare, even if these reductions are transitory. A recent review of canine enrichment in the shelter by Gunter and Feuerbacher (under review) [53] characterized a variety of interventions that can positively impact the lives of shelter-living dogs.

In addition to the previously mentioned in-shelter human interactions and fostering programs where the dog leaves the kennel and spends time with a person in an effort to improve their welfare, Gunter and Feuerbacher [53] suggested that dogs would benefit from more enriched living conditions while in the shelter, such as beds to lie upon and objects that they prefer to chew, such as soft, plush toys. With regards to social interaction with other dogs, Gunter et al. (in prep) [54] recently found that when dogs were provided three, 15 min conspecific sessions a day, their levels of Secretory Immunoglobin-A, an immune function antibody, were lower when compared to days when no dog contact was provided, suggesting that time with other dogs could also be beneficial to their welfare.

### 4.4. Individual Shelter Differences

While we were able to detect an overall effect of the intervention on cortisol values across our study, we were also able to detect overall shelter differences as well as variability in how the intervention affected dogs at the individual shelters. For example, cortisol values of dogs at SCRAPS were, on average, lower than those at other shelters. Differences in the magnitude of the intervention’s impact were also seen. Specifically, the arousing effect of the field trips, as indicated by increased C/C ratios, was detected most strongly at SCRAPS and RCACP, likely because of their lower in-shelter cortisol values. Yet, dogs at DACC and FCAS that also had higher afternoon cortisol values during field trips did not have statistically significant differences in pre- and post-outing comparisons.

We also found that cortisol values obtained at SCRAPS and RCACP failed to demonstrate evidence of a circadian rhythm effect: both morning and afternoon cortisol values were similar. One shelter, DACC, showed the strongest potential evidence of reduction across the day; however, a possible explanation for this reduction may be related to their operating hours. While SCRAPS and RCACP remained open until the early evening, DACC closed to potential adopters, volunteers, and most staff by 3:30 p.m. Hewison, Wright, Zulch, and Ellis [55] found that closing a shelter to the public during afternoons led to reductions in noise, increases in dogs’ sedentary behavior, along with decreases in locomotor and stereotypic behaviors. Thus, the reduction in cortisol across the day at DACC may be related to an absence of late-day stressors. If so, it may be possible that shelter dogs’ cortisol levels could decrease across the day, but the stimulating nature of human traffic at adoption facilities in the afternoons and early evenings may be preventing this from occurring.

### 4.5. Distal Effects on Welfare

While we have not found evidence here for the proximate welfare advantages of brief outings, it is possible that short-term outings may benefit shelter dogs in ways we did not measure. Outings with a volunteer, staff person, or member of the public may support adoptions and foster recruitment efforts by increasing the visibility of adoptable dogs in the community, which could benefit dogs’ ultimate welfare by enabling them to permanently leave the shelter and live in a home. In an ongoing investigation evaluating the deployment and implementation of short-term outing programs in animal shelters, preliminary analyses indicate that over 5% of dogs that experience a field trip are adopted by the person taking them on that trip. Future studies that explore these distal effects would aid shelters in the evidence-based care they provide to homeless dogs by identifying which enrichment interventions improve dogs’ daily experiences and which programs are associated with decreased lengths of stay and increased likelihood of adoption.

### 4.6. Limitations

When considering the limitations of our study, not all dogs at these shelters were eligible to participate, particularly dogs that were unable to walk on leash, fearful of the urine collection tools, or deemed unsafe to handle by shelter staff. While efforts were made to conduct this study at shelters that were representative of animal sheltering in the US, the four participating shelters were open-admission facilities. It is possible that facilities with more managed intake policies and lower intake numbers may have dogs in different living conditions that would respond differently than the findings reported here.

Additionally, the individuals taking dogs on short-term outings, though they were most often shelter staff and volunteers and members of the research team, did vary in their dog knowledge and handling skills, which may have affected the stress that dogs experienced during their field trips. Additionally, it is possible that a dog’s familiarity with the individual providing the short-term outing may further impact its experience [56]. However, it is worth noting that Gunter et al. [32] showed a consistent reduction in stress when dogs left the shelter during one and two nights of temporary fostering, which were provided by members of the public and shelter volunteers.

## 5. Conclusions

This study demonstrates that shelter dogs’ urinary cortisol concentrations increased during the afternoon of a short-term outing, even when accounting for their activity throughout the study. During the afternoon of the intervention, dogs’ high-intensity activity increased, and low-intensity activity decreased. These changes in cortisol and activity, however, were temporary, and both returned to pre-outing levels by the following day.

The magnitude of the intervention’s effect on cortisol and activity varied between the participating shelters with values differing amongst shelters as well as at this study’s various timepoints, suggesting that the living conditions at these facilities also influence dog welfare. In all, our findings indicate that short-term outings as tested here do not provide the reductions in stress achieved with temporary fostering in a home. Nevertheless, it is possible that outings of this type benefit shelter dogs’ distal welfare by increasing adoption prospects within the community and should be investigated further to understand this effect.

## Figures and Tables

**Table 1:** Shelter admission type and state where located, canine intake for the prior year, and number of dogs, complete sequences, and sample details.

Shelter	State	Prior Year Canine Intake	Subject Dogs	Complete Sequences *	Samples Collected	Samples Removed **
SCRAPS	WA	3074	41	36	204	8
FCAS	GA	6321	40	34	200	7
DACC	MI	4063	41	36	205	6
RCACP	VA	2027	42	37	210	6

Note. * Complete sequences are dogs in which all five collection timepoints were obtained and used in the analysis. ** Samples were removed from data analysis when urinary cortisol:creatinine ratio values were three standard deviations above the shelter’s mean.

**Table 2:** Experimental timeline for urine collection.

Window	Morning Collection (7:00–9:30 a.m.)	Short-Term Outing (11:00 a.m.–3:00 p.m.)	Afternoon Collection (3:30–6:00 p.m.)
Day 1			Sample 1
Day 2	Sample 2	Outing: ~2.5 h	Sample 3
Day 3	Sample 4		Sample 5

Note. Due to the time window in which outings could occur, collection for Sample 3 commenced two hours after the dog had returned from its outing. Additionally, collection for Sample 5 was attempted at a time that was between dogs’ collection times for Samples 1 and 3.

**Table 3:** Activity level categorization as based on each quintile’s magnitude-per-minute thresholds.

Activity Level	Low Threshold	High Threshold
Q1 (Low Activity)	0.0001	548.14
Q2	548.14	1217.75
Q3	1217.93	2475.77
Q4	2475.82	3058.04
Q5 (High Activity)	3058.04	8102.00

**Table 4:** By shelter, individuals taking dogs on short-term outings that were members of the public, that were female; outings that included more than one person participating; outings where no additional people interacted with the dog; and outings where 1–5 additional people interacted with the dog.

Shelter	% Public of Outing Participants *	% Female of Outing Participants	% Outings with >1 People on Outing	% Outings where No Additional People Interacted with Dog	% Outings Where 1–5 Additional People Interacted with Dog **
SCRAPS	31.70	78.05	53.66	21.95	63.41
FCAS	80.00	92.50	52.50	40.00	55.00
DACC	4.88	82.93	43.90	48.78	50.00
RCACP	35.71	92.86	42.86	30.95	43.90

Note. * Individuals responsible for taking dogs on short-term outings that were not members of the public were either shelter staff and volunteers or part of the research team. ** Outings in which more than five people interacted with the dog account for the remaining percentage of outings not reported here.

**Table 5:** Mean cortisol:creatinine ratio values, standard errors, *F* test statistics, and *p* values for five timepoints before and after a short-term outing at four US animal shelters.

			Pre-Field Trip						Post-Field Trip					
			Afternoon before (Day 1)		Morning before (Day 2)		Afternoon of Field Trip (Day 2)		Morning after (Day 3)		Afternoon after (Day 3)			
	Average		1		2		3		4		5		Test Statistics	
Shelter	M	SE	M	SE	M	SE	M	SE	M	SE	M	SE	*F*	*p*
SCRAPS	24.58	2.55	24.02	3.00	23.12^b^	2.93	29.59 ^a,b^	3.00	23.30	2.85	22.87^a^	2.86	2.79	0.026
FCAS	32.00	2.25	30.81	2.60	32.11	2.68	34.32	2.72	35.71	2.71	32.05	2.70	1.58	0.177
DACC	34.81	2.03	31.09 ^c^	2.46	36.81	2.52	35.59	2.47	37.89 ^c^	2.43	32.66	2.46	3.03	0.017
RCACP Overall	31.07	2.10	30.06 ^d^ 28.99 ^h^	2.48 1.26	28.60 ^e^ 30.16	2.55 1.33	36.99 ^d,e,f,g^ 34.12 ^h,i^	2.60 1.39	30.46 ^g^ 31.84	2.52 1.31	29.26 ^f^ 29.21 ^i^	2.46 1.29	4.00 6.29	0.003 <0.001

Note. All comparisons (shared letters: a–i) are significant at *p* = 0.05 or less except for comparisons b (*p* = 0.051) and g (*p* = 0.068).

## Data Availability

The data presented in this study are openly available at the Dataverse, the Arizona State University Library’s Research Data Repository [https://doi.org/10.48349/ASU/XPISIW].
